# Numerical treatment of the radiated and dissipative power-law nanofluid flow past a nonlinear stretched sheet with non-uniform heat generation

**DOI:** 10.1038/s41598-023-49547-7

**Published:** 2023-12-20

**Authors:** M. M. Khader, Ahmed M. Megahed, A. Eid

**Affiliations:** 1 Department of Mathematics and Statistics, College of Science, Imam Mohammad Ibn Saud Islamic University (IMSIU), Riyadh, 11566 Saudi Arabia; 2 Department of Mathematics, Faculty of Science, Benha University, Benha, Egypt; 3 Department of Physics, College of Science, Imam Mohammad Ibn Saud Islamic University (IMSIU), Riyadh, 11566 Saudi Arabia; 4 Department of Astronomy, Faculty of Science, Cairo University, Giza, Egypt

**Keywords:** Nanoscience and technology, Physics

## Abstract

The main aim of this paper is to investigate the effect of non-uniform heat generation and viscous dissipation on the boundary layer flow of a power-law nanofluid over a nonlinear stretching sheet. Within the thermal domain, the analysis considers both thermal radiation and variable thermal conductivity. Through **the use of** similarity transformations, the governing boundary layer equations are transformed into a system of ODEs. The spectral collocation method (SCM) with shifted Vieta-Lucas polynomials (VLPs) is implemented to give an approximate expression for the derivatives and then use it to **numerically solve** the proposed system of equations. By employing this technique, the system of ODEs is converted into a system of nonlinear algebraic equations. The dimensionless temperature, concentration, and velocity are graphically presented and analyzed for various values of the relevant governing parameters. Through the presented graphical solutions, we can see that the main outcomes indicate that an increase in the power law index, thermal conductivity parameter, and radiation parameter leads to a noticeable decrease in the local Nusselt number, with reductions of around 0.05 percent, 0.23 percent, and 0.11 percent, respectively. In contrast, the Prandtl parameter demonstrates an opposing effect, elevating the local Nusselt number by about 0.1 percent. We validated the accuracy of the numerical solutions by comparing them in some special cases with existing literature.

## Introduction

In recent decades, there has been increasing interest on the part of researchers to study **the** technological applications of non-Newtonian power-law fluids due to their diverse potential uses^1^. These materials are notably utilized in various fields, including geophysics, cosmetic processes, oil reservoir engineering, paper production, bioengineering **and** chemical, polymer solutions, and nuclear industries, among others. Moreover, the response of all non-Newtonian materials to shear cannot be precisely predicted using a single constitutive relationship. As a result, researchers have introduced multiple models of non-Newtonian fluids to facilitate discussions about their diverse characteristics and properties. The necessity for employing multiple models stems from the fact that different non-Newtonian fluids demonstrate distinctive and intricate behaviors under various flow conditions. These diverse models aid in enhancing our comprehension and describing the distinct rheological properties of these fluids. These endeavors play a significant role in advancing our understanding and application of non-Newtonian fluids in various **industrial and** scientific domains. Among these models, the power-law model stands out as the simplest one, representing the commonly observed behaviors of fluids, such as shear thinning and shear thickening. Schowalter^2^ was the one who applied the concept of the boundary layer to power-law fluids (PLFs). Fluids with power-law flow characteristics are commonly encountered in various practical applications, including blood, polymers, molten plastics, foodstuffs, and more. PL non-Newtonian fluids present numerous benefits in diverse industrial and practical applications when contrasted with Newtonian fluids. These advantages arise primarily from the presence of the flow behavior index in the PLFs, which enables a broad spectrum of viscosity adjustments depending on the shear rate. Through careful selection of the flow behavior index, engineers can customize the fluid’s viscosity to suit particular application needs, making it an invaluable asset in processes that demand precise control over flow characteristics^3^. Several studies focusing on flows described by the power-law model have been referenced in works^4–6^.

Lately, nanotechnology’s exploration involving nanofluids has garnered significant interest due to its extensive applications across various engineering and technological fields. A nanofluid consists of particles with sizes smaller than 100 nanometers that are dispersed within the base fluid^7^. Nanofluids represent the next evolutionary step in heat transfer liquids, presenting intriguing prospects for improving heat transfer efficiency when compared to pure liquids. Nanofluids find diverse uses in hybrid-powered engines, chemical catalytic reactors, and other applications. This is because traditional fluids like **water and oil** are generally considered inefficient heat transfer fluids due to their low thermal conductivity^8^. One highly dependable method to improve the thermal conductivity of such fluids involves incorporating nanoparticles with relatively higher conductivities into the base fluid^9^. The non-Newtonian behavior exhibited by fluids containing nanoparticles, known as power law nanofluid flow, presents numerous real-life applications. These applications span biomedical uses, cooling for electronic devices, solar thermal systems, and food processing. The distinctive rheological properties of the PL nanofluids are harnessed in these applications to tackle challenges associated with heat transfer, lubrication, and thermal management across diverse industrial and scientific settings. The findings have confirmed that the presence of nanoparticles in different types of nanofluids can indeed augment the heat transport mechanism and the thermal conductivity of the fluid. Further details and related investigations can be found in the listed references^10–15^. Enhancing heat transfer in heat exchangers, **double-plane** windows, electronic cooling, and similar applications are of utmost importance for energy conservation. Several studies have been conducted to investigate nanofluids and their potential applications in addressing this concern^16–18^.

Indeed, the majority of nonlinear differential equations lack exact solutions, necessitating the use of numerical and approximate methods as the primary approaches to solving such ordinary differential equations (ODEs)^19,20^. The SCM is an approximation method utilized to solve numerically ODEs. This technique gives the approximate solution by summing up basis functions and determining their coefficients by collocating the differential equation at a limited number of collocation points^21–23^. Among the variety of base functions available for use are the orthogonal VLPs. Utilizing the SCM with VLPs comes with the benefit of their remarkable convergence properties. The accuracy of the solution improves rapidly with an increase in the number of collocation points. Moreover, VLPs exhibit good stability properties, making them suitable for solving differential equations that are stiff or have rapidly varying solutions. In addition, the VLPs have the added advantage of having a closed-form expression, which simplifies their computation and manipulation^24,25^.

The novelty, purpose, and drive behind this study become evident when examining the importance of non-Newtonian behavior in the industry, and modern technology. No prior research has delved into the amalgamation of the power-law model with viscous dissipation originating from non-uniform heat generation on a nonlinear stretched sheet. Therefore, this paper seeks to utilize the Vieta-Lucas spectral collocation approach to numerically solve the non-Newtonian power-law model and **consider** elements like viscous dissipation, thermal radiation, and non-uniform heat generation within the framework of a nonlinear stretching sheet. In light of these advancements, one could ponder the potential future directions that could further explore the applications and behaviors of PL nanofluid flow. To achieve this main aim, we will apply the SCM based on VLPs as a basis to convert the resulting system of ODEs to a system of algebraic equations. This system is considered a constrained optimization problem and optimized to get the unknown coefficients of the series of the solution. This connection of the two well-known techniques will be called “the shifted Vieta-Lucas collocation optimization method (SVLCOM)”.

## Mathematical formulation of the problem

We are examining the continuous 2-dimensional movement of a non-compressible nanofluid with power-law properties, flowing over a stretching sheet with a nonlinear shape. The components of velocity *u* and *v* are respectively aligned parallel and $\perp$ to the surface. The flow is induced by two opposite and equal forces along the *x*-axis, causing the sheet to stretch at a velocity $U_{w}=cx^{m}$, where *c* and *m* are positive real numbers, while the origin remains stationary. The origin is positioned at the slit, where the sheet is pulled through the fluid medium. The stretching sheet is held at different temperatures $T_{w}=T_{\infty }+A\,x^{r}$, and concentrations $C_{w}=C_{\infty }+B\,x^{s}$ for some constants *A* and *B*; where $T_{\infty }$ and $C_{\infty }$, are constants and uniforms. Furthermore, the constants *m*, *r*, and *s* are fixed and can be determined to ensure the satisfaction of the similarity solution. Additionally, it is postulated that the power-law nanofluid is influenced by the thermal radiation phenomenon, aligning with the presence of non-uniform heat generation. Further, the nanofluid’s thermal conductivity $\kappa$ is considered to vary during its motion, and this variation follows a linear temperature dependence. The relationship with temperature can be expressed as follows^26^:

1 $$\begin{aligned} \kappa =\kappa _{\infty }\left( 1+\varepsilon \left( \frac{T-T_{\infty }}{T_{w}-T_{\infty }}\right) \right) . \end{aligned}$$

**Figure 1:**
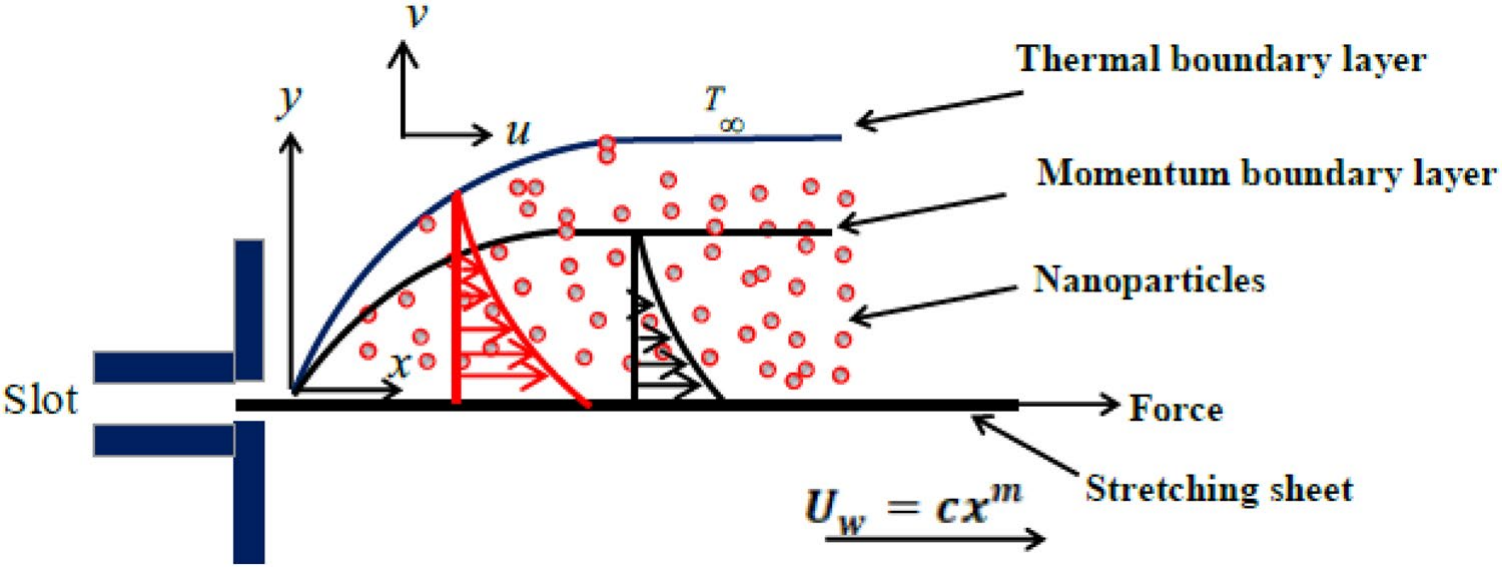
Description of the proposed power law nanofluid model.

In the given context, $\kappa _{\infty }$ represents the thermal conductivity at a distance from the sheet surface. In the energy equation, we take into account the viscous dissipation based on the power-law model, thermal radiation effects, Brownian motion of nanoparticles, and thermophoresis phenomena. Figure 1 provides a visual representation depicting the flow of the nanofluid induced by a nonlinear stretching sheet. The following equations represent the two-dimensional flow of the PL nanofluid, taking into account all the previously mentioned assumptions $\left( \nabla = \left( \frac{\partial }{\partial x},\frac{\partial }{\partial y}\right) \right)$^27,28^:

2 $$\begin{aligned}{} & {} \nabla .(u,v)=0, \end{aligned}$$

3 $$\begin{aligned}{} & {} \rho \left( (u,v).\nabla \,u\right) =\mu \frac{\partial }{\partial y}\left[ \left( -\frac{\partial u}{\partial y} \right) ^{n-1}\frac{\partial u}{\partial y}\right] , \end{aligned}$$

4 $$\begin{aligned}{} & {} \rho c_{p}\left( (u,v).\nabla \,T\right) =\frac{\partial }{\partial y}\Big (\kappa \frac{\partial T}{\partial y}\Big )+\rho c_{p}\tau \left( D_{B}\frac{\partial T}{\partial y}\frac{\partial C}{\partial y}+\frac{D_{T}}{T_{\infty }} \left( \frac{\partial T}{\partial y}\right) ^{2}\right) +\mu \left( -\frac{\partial \,u}{\partial y} \right) ^{n+1}+q^{'''}-\frac{\partial q_{r}}{\partial y}, \end{aligned}$$

5 $$\begin{aligned}{} & {} (u,v).\nabla \,C=D_{B} \frac{\partial ^{2} C}{\partial y^{2}}+\frac{D_{T}}{T_{\infty }}\frac{\partial ^{2} T}{\partial y^{2}}. \end{aligned}$$

When the power-law index $n<1$, the fluid is categorized as a pseudo-plastic material, exhibiting shear-thinning properties. If $n>1$, it is referred to as a dilatant substance, showing shear-thickening characteristics. Finally, when $n=1$, the fluid behaves as a Newtonian fluid. Likewise, the radiative heat flux, denoted by $q_{r}$, plays a crucial role in our model. It is governed by the following relationship^29^:

6 $$\begin{aligned} q_{r}=-\frac{4\sigma ^{*}}{3k^{*}}\frac{\partial T^{4}}{\partial y}. \end{aligned}$$

The Stefan-Boltzmann constant, denoted by $\sigma ^{*}$, and $k^{*}$, are constants in this formula of $q_{r}$. These characteristics have a significant impact on the radiative heat flux and its interactions with the system. Also, we suppose that the temperature difference within the nanofluid flow allows expressing the term $T^{4}$ as a linear combination of temperatures. By utilizing Taylor’s series and considering only terms of low order, we arrive at the following formulation^30^:

7 $$\begin{aligned} T^{4}\cong -3T^{4}_{\infty }\left( 1-\frac{4T}{3T_{\infty }}\right) . \end{aligned}$$

Below are the explanations for the dimensionless boundary conditions:

8 $$\begin{aligned}{} & {} v=0, \quad u=U_{w}=c\,x^{m}, \quad T=T_{w}=T_{\infty }+A\,x^{r}, \quad C=C_{w}=C_{\infty }+B\,x^{s}, \quad at \quad y=0, \end{aligned}$$

9 $$\begin{aligned}{} & {} u\rightarrow 0, \quad \quad C\rightarrow C_{\infty }, \quad \quad T\rightarrow T_{\infty } \quad \quad \text{ as }, \quad \quad y \rightarrow \infty . \end{aligned}$$

The similarity transformations, employed in terms of $\theta$, *f* and $\phi$ as well as the similarity variable $\eta$ to solve the governing equations, are outlined as follows^27^:

10 $$\begin{aligned} \eta= & {} \left[ \left( \frac{\mu }{\rho }\right) ^{\frac{-1}{n+1}}x^{\frac{m(2-n)-1}{n+1}}c^{\frac{2-n}{n+1}} \right] y, \quad \quad \quad \psi (\eta )=\left( \frac{\mu }{\rho }\right) ^{\frac{1}{n+1}}x^{\frac{m(2n-1)+1}{n+1}}c^{\frac{2n-1}{n+1}}f(\eta ), \end{aligned}$$

11 $$\begin{aligned} \phi (\eta )= & {} \frac{C-C_{\infty }}{C_{w}-C_{\infty }}, \quad \quad \quad \quad \theta (\eta )=\frac{T-T_{\infty }}{T_{w}-T_{\infty }}. \end{aligned}$$

In the last relations, $\psi$ fulfills the continuity equation (2) and is characterized by:

12 $$\begin{aligned} u=\frac{\partial \psi }{\partial y},\quad \quad \quad \quad v=-\frac{\partial \,\psi }{\partial x}. \end{aligned}$$

Additionally, we have considered the particular form of non-uniform heat generation $q^{'''}$, which was introduced earlier by Abo-Eldahab and El Aziz^31^, in our analysis. This form is represented as follows:

13 $$\begin{aligned} q^{'''}=\frac{ \kappa U_{w}}{\nu x}\left[ A^{*}(T_{w}-T_{\infty })e^{-\eta }+B^{*}(T-T_{\infty })\right] . \end{aligned}$$

Here, we must mention that when the coefficients $A^{*}$ and $B^{*}$ are both positive, it indicates internal heat generation. Conversely, if both $A^{*}$ and $B^{*}$ are negative, it suggests internal heat absorption.

By utilizing similarity transformations defined in Eqs. (10, 11), the continuity equation (2) is satisfied exactly, and Eqs. (3, 4) are transformed into the following expressions:

14 $$\begin{aligned}{} & {} nf^{\prime \prime \prime }\left( -f^{\prime \prime }\right) ^{n-1}-mf^{\prime 2}+\Upsilon _{m,n}\,ff^{\prime \prime }=0, \end{aligned}$$

15 $$\begin{aligned}{} & {} \frac{1}{{\text {Pr}}}\left( (1+R+\varepsilon \,\theta )\theta ^{\prime \prime }+\varepsilon \,\theta ^{\prime 2}\right) -r\,\theta f^{\prime }+\Upsilon _{m,n}\,f\theta ^{\prime }+Ec\left( -f^{\prime \prime }\right) ^{n+1}\nonumber \\{} & {} \quad +(1+\varepsilon \,\theta )\left( \gamma ^{*}e^{-\eta }+\gamma \,\theta \right) +\Lambda _{t}\,\theta ^{\prime 2}+\Lambda _{b}\,\theta ^{\prime } \phi ^{\prime }=0, \end{aligned}$$

16 $$\begin{aligned}{} & {} \phi ^{\prime \prime }+\frac{\Lambda _{t}}{\Lambda _{b}}\theta ^{\prime \prime }-s\,\phi \,f^{\prime }+Sc\,\Upsilon _{m,n}\,f\phi ^{\prime }=0, \end{aligned}$$

where $\Upsilon _{m,n}=\frac{m(2n-1)+1}{n+1}$. The modified boundary conditions are defined in the following form:

17 $$\begin{aligned}{} & {} f(0)=0, \quad \quad \quad \quad f^{\prime }(0)=\theta (0)=\phi (0)=1, \end{aligned}$$

18 $$\begin{aligned}{} & {} f^{\prime }\rightarrow 0, \quad \quad \quad \theta \rightarrow 0, \quad \quad \quad \phi \rightarrow 0, \quad \quad \quad as \quad \quad \eta \rightarrow \infty . \end{aligned}$$

Below is a concise overview of the explanations for each parameter governing the aforementioned system **of momentum**, energy, and concentration:

19 $$\begin{aligned} R= & {} \frac{16\sigma ^{*} T_{\infty }^{3}}{3\kappa _{\infty } k^{*}}, \quad Ec=\frac{U_{w}^{2}}{c_{p}(T_{w}-T_{\infty })}, \quad Pr=\frac{\rho \,c_{p}}{\kappa }\left( x^{(3m-1)(n-1)}c^{3(n-1)}(K/\rho )^{2}\right) ^{\frac{1}{n+1}}, \quad \,Sc=\frac{\nu }{D_{B}}, \end{aligned}$$

20 $$\begin{aligned} \Lambda _{b}= & {} \frac{\tau D_{B}\left( C_{w}-C_{\infty }\right) }{\nu }, \quad \quad \Lambda _{t}=\frac{\tau D_{T}\left( T_{w}-T_{\infty }\right) }{\nu T_{\infty }}, \quad \quad \gamma ^{*}=\frac{\kappa _{\infty } A^{*}}{\mu c_{p}}, \quad \quad \gamma =\frac{\kappa _{\infty } B^{*}}{\mu c_{p}}. \end{aligned}$$

Further, based on the definitions of the preceding parameters, it can be deduced that a similarity solution exists when $m=\frac{1}{3}$ and $r=s=\frac{2}{3}$.

## Engineering and industrial quantities

Using the similarity transformation, we can derive the local Sherwood number $Sh_{x}$, local Nusselt number $Nu_{x}$, and local skin-friction coefficient $Cf_{x}$ as follows:

21 $$\begin{aligned} Sh_{x}=-Re_{x}^{\frac{1}{n+1}}\phi ^{\prime }(0), \quad Cf_{x}=2(-1)^{n}\,Re_{x}^{\frac{-1}{n+1}} \left( f^{\prime \prime }(0)\right) ^{n}, \quad Nu_{x}=-Re_{x}^{\frac{1}{n+1}}(1+R)\theta ^{\prime }(0). \end{aligned}$$

where $Re_{x}=\frac{U_{w}^{2-n}x^{n}}{\nu }$ is the local non-Newtonian Reynolds number.

## Basic concepts on the shifted VLPs

We are going to research a class of orthogonal polynomials, which lies at the heart of our work and they are necessary to reach our goal. With the help of their recurrence relations and analytical formulae, we can generate and construct a new family of these polynomials that will be known as VLPs.

The VLPs; $\textrm{VL}_{m}(x)$ of degree $m \in {\mathbb {N}}_{0}$ is obtained through the following formula^32^:

$$\begin{aligned} \textrm{VL}_{m}(x)=2 \cos (m \chi ), \quad \,\,\,\, \chi =\cos ^{-1}\,\left( 0.5\,x\right) ,\quad \,\,\,\, \chi \in [0, \pi ], \quad \,\,\,\, -2\le \,x\le 2. \end{aligned}$$

These VLPs satisfy the following recurrence formula:

$$\begin{aligned} \textrm{VL}_{m}(x)=x \textrm{VL}_{m-1}(x)-\textrm{VL}_{m-2}(x), \,\,\,\,\quad m=2,3, \ldots , \,\,\,\,\quad \textrm{VL}_{0}(x)=2, \,\,\,\,\quad \textrm{VL}_{1}(x)=x. \end{aligned}$$

Now, by utilizing the linear transformation $x=4\eta -2$, we can generate a new class of orthogonal polynomials of VLPs but on the interval [0, 1], and it will be denoted by $\textrm{VL}_{m}^{s}(\eta )$ and can be obtained from the formula $\textrm{VL}_{m}^{s}(\eta )=\textrm{VL}_{m}(4\eta -2).$

The polynomials $\textrm{VL}_{m}^{s}(\eta )$ satisfy the recurrence relation defined as:

$$\begin{aligned} \textrm{VL}_{m+1}^{s}(\eta )=(4\,\eta -2) \textrm{VL}_{m}^{s}(\eta )-\textrm{VL}_{m-1}^{s}(\eta ), \,\,\,\,\quad \quad m=1,2, \ldots , \end{aligned}$$

where,  $\textrm{VL}_{0}^{s}(\eta )=2, \,\, \textrm{VL}_{1}^{s}(\eta )=4\eta -2.$ Also, we find $\textrm{VL}_{m}^{s}(0)=2(-1)^{m}$ and $\textrm{VL}_{m}^{s}(1)=2,\,\,m=0,1,2,....$

The analytical formula for $\textrm{VL}_{m}^{s}(\eta )$ is given by:

$$\begin{aligned} \textrm{VL}_{m}^{s}(\eta )=2 m \sum _{j=0}^{m}(-1)^{j} \frac{4^{m-j} \Gamma (2 m-j)}{\Gamma (j+1) \Gamma (2 m-2 j+1)} \,\eta ^{m-j}, \quad \quad \,\,\,\, m=2,3, \ldots . \end{aligned}$$

Let $g(\eta )$ be a function in the space $L^2 [0,1]$, then by using the shifted VLPs, this function can be expressed and approximated in terms of the first $(m+1)$-terms of $\textrm{VL}_{m}^{s}(\eta )$ as follows:

22 $$\begin{aligned} g(\eta )\approx \,g_{m}(\eta )=\sum _{j=0}^{m} \alpha _{j} \textrm{VL}_{j}^{s}(\eta ), \end{aligned}$$

where $\alpha _{j}$ are constants that we should evaluate with the help of the orthogonality condition of these polynomials.

## An approximate of $g^{(n)}_{m}(\eta )$

In this section, we present an approximate formula of $g^{(n)}_{m}(\eta )$, and state some notes and formulas concerning the convergence analysis by computing the error estimate of that approximation.

### Theorem 1

The *n*-order derivative for the function $g_{m}(\eta )$ which is defined in Eq. (22) can be estimated by the following formula^33^:

23 $$\begin{aligned} g^{(n)}_{m}(\eta )=\sum _{j=n}^{m} \sum _{k=0}^{j-n} \alpha _{j}\,\frac{(-1)^{k}\,4^{j-k} (2\,j)\,(2 j-k-1)!\,(j-k)!}{k!\,(2 j-2 k)!\,(j-k-n)!}\, \eta ^{j-k-n}. \end{aligned}$$

### Theorem 2

^34^ Assume that $g(\eta )\in \,L_{\tilde{{\textbf {w}}}}^{2}\,[0,1]$ w.r.t. the weight function $\tilde{{\textbf {w}}}(\eta )=\frac{1}{\sqrt{\eta -\eta ^2}}$, and $|g''(\eta )|\le \lambda$, for some constant $\lambda$. Then the series (22) converges uniformly to the function $g(\eta )$ as $m \rightarrow \infty$. In addition, we have the following facts:

1. The coefficients’s series in Eq. (22) are bounded, i.e.
  $$\begin{aligned} \left| \alpha _{j}\right| \le \frac{\lambda }{4 j \left( j^{2}-1 \right) }, \quad \quad j>2. \end{aligned}$$
2. The error estimate norm $\left( L_{\tilde{{\textbf {w}}}}^{2}\,[0,1]-norm \right)$ can be defined as follows:
  $$\begin{aligned} \left\| g(\eta )-g_{m}(\eta )\right\| _{\tilde{{\textbf {w}}}}<\frac{L}{12\,\sqrt{m^{3}}}. \end{aligned}$$
3. If $g^{(m)}(\eta )\in \,C[0,1]$, then the absolute error bound holds:
  $$\begin{aligned} \left\| g(\eta )-g_{m}(\eta )\right\| \le \frac{\Delta \, \Pi ^{m+1}}{(m+1) !} \sqrt{\pi },\quad \quad \Delta =\max _{\eta \in [0,1]}\,g^{(m+1)}(\eta ), \,\,\, \hbox {and} \,\,\, \Pi =\max \left\{ 1-\eta _{0}, \eta _{0}\right\} . \end{aligned}$$

## Procedure solution using SVLCOM

In this section, we are going to solve numerically the given problem (14–16) by implementing the shifted Vieta-Lucas collocation optimization method, through the following steps^24^:

1. We approximate the solution of the problem (14–16) in the following form as a finite series of shifted VLPs:
  24 $$\begin{aligned} f_{N}(\eta )=\sum _{j=0}^{N}\alpha _{j}\,\textrm{VL}_{j}^{s}(\eta ),\quad \quad \,\,\, \theta _{N}(\eta )=\sum _{j=0}^{N}\beta _{j}\,\textrm{VL}_{j}^{s}(\eta ), \quad \quad \,\,\,\phi _{N}(\eta )=\sum _{j=0}^{N}\delta _{j}\,\textrm{VL}_{j}^{s}(\eta ). \end{aligned}$$
2. We connect between (24) and the approximation (23) in the given model (14–16) to get:
  25 $$\begin{aligned}{} & {} n\,\Big (f^{(3)}_{N}(\eta )\Big )\left( -f^{(2)}_{N}(\eta )\right) ^{n-1}-m\,\left( f^{(1)}_{N}(\eta )\right) ^{2}+ \Upsilon _{m,n}\,\left( f_{N}(\eta )\right) \left( f^{(2)}_{N}(\eta )\right) =0, \end{aligned}$$
  26 $$\begin{aligned}{} & {} \frac{1}{{\text {Pr}}}\Big (\left( 1+R+\varepsilon \,\theta _{N}(\eta )\right) \left( \theta ^{(2)}_{N}(\eta )\right) +\varepsilon \,\left( \theta ^{(1)}_{N}(\eta )\right) ^{2}\Big )-r\,\left( \theta _{N}(\eta )\right) \left( f^{(1)}_{N}(\eta )\right) \nonumber \\{} & {} \quad +\Upsilon _{m,n}\,\left( f_{N}(\eta )\right) \left( \theta ^{(1)}_{N}(\eta )\right) +Ec\left( -f^{(2)}_{N}(\eta )\right) ^{n+1}+\left( 1+\varepsilon \,\theta _{N}(\eta )\right) {\textbf {.}}\nonumber \\{} & {} \quad \left( \gamma ^{*}e^{-\eta }+\gamma \,\theta _{N}(\eta )\right) +\Lambda _{t}\,\left( \theta ^{(1)}_{N}(\eta )\right) ^{2}+\Lambda _{b}\, \left( \theta ^{(1)}_{N}(\eta )\right) \left( \phi ^{(1)}_{N}(\eta )\right) =0, \end{aligned}$$
  27 $$\begin{aligned}{} & {} \phi ^{(2)}_{N}(\eta )+\frac{\Lambda _{t}}{\Lambda _{b}}\,\left( \theta ^{(2)}_{N}(\eta )\right) -s\,\left( \phi _{N}(\eta )\right) \left( f^{(1)}_{N}(\eta )\right) +Sc\,\Upsilon _{m,n}\,\left( f_{N}(\eta )\right) \left( \phi ^{(1)}_{N}(\eta )\right) =0. \end{aligned}$$
3. We collocate the system (25–27) at $N-2$ of points $\eta _{k}, k=0,1,2,\ldots ,N-3$ as follows:
  28 $$\begin{aligned}{} & {} n\,\Big (f^{(3)}_{N}(\eta _{k})\Big )\left( -f^{(2)}_{N}(\eta _{k})\right) ^{n-1}-m\,\left( f^{(1)}_{N}(\eta _{k})\right) ^{2}+ \Upsilon _{m,n}\,\left( f_{N}(\eta _{k})\right) \left( f^{(2)}_{N}(\eta _{k})\right) =0, \end{aligned}$$
  29 $$\begin{aligned}{} & {} \frac{1}{{\text {Pr}}}\Big (\left( 1+R+\varepsilon \,\theta _{N}(\eta _{k})\right) \left( \theta ^{(2)}_{N}(\eta _{k})\right) +\varepsilon \,\left( \theta ^{(1)}_{N}(\eta _{k})\right) ^{2}\Big ) -r\,\left( \theta _{N}(\eta _{k})\right) \left( f^{(1)}_{N}(\eta _{k})\right) \nonumber \\{} & {} \quad +\Upsilon _{m,n}\,\left( f_{N}(\eta _{k})\right) \left( \theta ^{(1)}_{N}(\eta _{k})\right) +Ec\left( -f^{(2)}_{N}(\eta _{k})\right) ^{n+1} +\left( 1+\varepsilon \,\theta _{N}(\eta _{k})\right) {\textbf {.}}\nonumber \\{} & {} \quad \left( \gamma ^{*}e^{-\eta _{k}}+\gamma \,\theta _{N}(\eta _{k})\right) +\Lambda _{t}\, \left( \theta ^{(1)}_{N}(\eta _{k})\right) ^{2}+\Lambda _{b}\, \left( \theta ^{(1)}_{N}(\eta _{k})\right) \left( \phi ^{(1)}_{N}(\eta _{k})\right) =0, \end{aligned}$$
  30 $$\begin{aligned}{} & {} \phi ^{(2)}_{N}(\eta _{k})+\frac{\Lambda _{t}}{\Lambda _{b}}\,\left( \theta ^{(2)}_{N} (\eta _{k})\right) -s\,\left( \phi _{N}(\eta _{k})\right) \left( f^{(1)}_{N}(\eta _{k})\right) +Sc\,\Upsilon _{m,n}\,\left( f_{N}(\eta _{k})\right) \left( \phi ^{(1)}_{N}(\eta _{k})\right) =0. \end{aligned}$$
  Also, we approximate the boundary conditions (17)-(18) at the finite interval $(0,\eta _{\infty })$ in **the following form:**
  31 $$\begin{aligned}{} & {} \sum _{j=0}^{N}2(-1)^j\,\alpha _{j}=0,\quad \,\,\, \sum _{j=0}^{N}\alpha _{j}\,\textrm{VL}_{j}^{s'}(0)=1,\quad \,\,\, \sum _{j=0}^{N}2(-1)^j\,\beta _{j}=1, \quad \,\,\, \sum _{j=0}^{N}2(-1)^j\,\delta _{j}=1, \end{aligned}$$
  32 $$\begin{aligned}{} & {} \sum _{j=0}^{N}a_{j}\,\textrm{VL}_{j}^{*'}(\eta _{\infty })=0,\quad \quad \quad \sum _{j=0}^{N}2\beta _{j}=0,\quad \quad \sum _{j=0}^{N}2\delta _{j}=0. \end{aligned}$$
4. We recognize the obtained system (28)-(32) as a constrained optimization problem by introducing the following cost functions (CFs):
  33 $$\begin{aligned} CF1= & {} \sum _{k=0}^{N}\Big |n\,\Big (f^{(3)}_{N}(\eta _{k})\Big ) \left( -f^{(2)}_{N}(\eta _{k})\right) ^{n-1}-m\,\left( f^{(1)}_{N}(\eta _{k})\right) ^{2} +\Upsilon _{m,n}\,\left( f_{N}(\eta _{k})\right) \left( f^{(2)}_{N}(\eta _{k})\right) \Big |, \end{aligned}$$
  34 $$\begin{aligned} CF2= & {} \sum _{k=0}^{N}\Big |\frac{1}{{\text {Pr}}}\Big (\left( 1+R+\varepsilon \, \theta _{N}(\eta _{k})\right) \left( \theta ^{(2)}_{N}(\eta _{k})\right) +\varepsilon \,\left( \theta ^{(1)}_{N}(\eta _{k})\right) ^{2}\Big )-r\, \left( \theta _{N}(\eta _{k})\right) \left( f^{(1)}_{N}(\eta _{k})\right) \nonumber \\{} & {} \quad +\Upsilon _{m,n}\,\left( f_{N}(\eta _{k})\right) \left( \theta ^{(1)}_{N} (\eta _{k})\right) +Ec\left( -f^{(2)}_{N}(\eta _{k})\right) ^{n+1} +\left( 1+\varepsilon \,\theta _{N}(\eta _{k})\right) {\textbf {.}}\nonumber \\{} & {} \quad \left( \gamma ^{*}e^{-\eta _{k}}+\gamma \,\theta _{N}(\eta _{k})\right) +\Lambda _{t}\, \left( \theta ^{(1)}_{N}(\eta _{k})\right) ^{2}+\Lambda _{b}\,\left( \theta ^{(1)}_{N}(\eta _{k})\right) \left( \phi ^{(1)}_{N}(\eta _{k})\right) \Big |, \end{aligned}$$
  35 $$\begin{aligned} CF3= & {} \sum _{k=0}^{N}\Big |\phi ^{(2)}_{N}(\eta _{k})+\frac{\Lambda _{t}}{\Lambda _{b}}\,\left( \theta ^{(2)}_{N}(\eta _{k})\right) -s\,\left( \phi _{N}(\eta _{k})\right) \left( f^{(1)}_{N}(\eta _{k})\right) \nonumber \\{} & {} \quad +Sc\,\Upsilon _{m,n}\, \left( f_{N}(\eta _{k})\right) \left( \phi ^{(1)}_{N}(\eta _{k})\right) \Big |, \end{aligned}$$
  and the constraints (Cons.)
  36 $$\begin{aligned} \hbox {Cons.}= & {} \Big |\sum _{j=0}^{N}2(-1)^j\,\alpha _{j}\Big | +\Big |\sum _{j=0}^{N}\alpha _{j}\,\textrm{VL}_{j}^{s'}(0)-1\Big |+\Big |\sum _{j=0}^{N}2(-1)^j \,\beta _{j}-1\Big |\nonumber \\{} & {} +\Big |\sum _{j=0}^{N}2(-1)^j\,\delta _{j}-1\Big | + \Big |\sum _{j=0}^{N}\,\alpha _{j}\,\textrm{VL}_{j}^{s'}(\eta _{\infty })\Big |+\Big |\sum _{j=0}^{N}2\beta _{j}\Big | +\Big |\sum _{j=0}^{N}2\delta _{j}\Big |. \end{aligned}$$
5. We solve numerically the constrained optimization problem (33–36) by implementing the Penalty Leap Frog procedure^35^ for the unknowns $\alpha _{j},\, \beta _{j},\, \delta _{j}, j= 0,1,2,\ldots ,N$. This led us to construct the approximate solution as given in the formula (24).

## Validation of the code’s accuracy

In this section, to evaluate the precision of the SCM combined with the shifted VLPs, a comparison is presented in Table 1 with the results obtained previously through a literature review. This table presents the results obtained in a particular scenario for different values of the power law index *n*. The outcomes closely align with the findings of previous research conducted by Andersson and Kumaran^36^ in a similar situation, thereby validating the accuracy of the solution.

**Table 1:** Comparison of values of skin friction coefficients $-f^{\prime \prime }(0)$ in connection with the prior work conducted by Andersson and Kumaran^36^ for different values of *n* when $m=1$.

*n*	Work in^36^	Current work
0.8	1.028400	1.028399854
0.9	1.011300	1.011298709
1.1	0.992400	0.992399014
1.3	0.984000	0.983899953
1.7	0.979501	0.979500892
1.8	0.979468	0.979467789

## Discussion of numerical results

**Figure 2:**
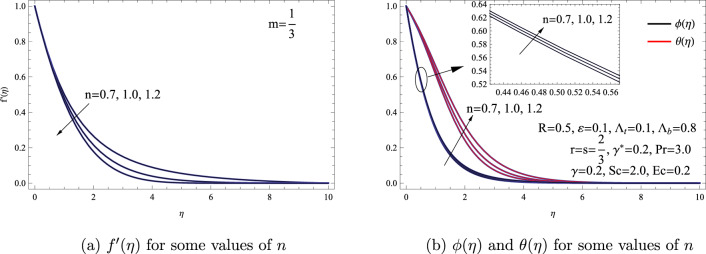
(**a**) $f'(\eta )$ for some values of *n*. (**b**) $\phi (\eta )$ and $\theta (\eta )$ for some values of *n*.

Our study primarily focuses on examining the transportation of thermal energy and mass within a **power-law** nanofluid flow over a nonlinear stretching surface. We also consider the effect of non-uniform heat generation, viscous dissipation, and thermal radiation as integral aspects of our analysis. In this section, we showcase the numerical outcomes utilizing the SCM employing shifted VLPs. We give a comprehensive portrayal of the flow and heat mass transportation, considering the impact of various physical factors. These factors include *n*, *R*, $\varepsilon$, $\Lambda _{t}$, $\Lambda _{b}$, $\gamma$, $\gamma ^{*}$, and *Ec*. During the numerical calculation, we kept the relevant parameters constant at a value of $n=1.2, R=0.5, \varepsilon =\Lambda _{t}=0.1, \gamma =\gamma ^{*}=Ec=0.2, Pr=3.0, Sc=2.0,$ and $\Lambda _{b}=0.8$ for the analysis. Figure 2 illustrates how variations in *n* influence the dimensionless concentration $\phi (\eta )$, dimensionless velocity $f'(\eta )$, and dimensionless temperature $\theta (\eta )$. The graphs in Figure 2 indicate that as *n* increases, the velocity flow field diminishes, resulting in flow occurring primarily near the surface. Conversely, the concentration and thermal fields exhibit an opposite trend. Physically, an elevation in the power-law index corresponds to an amplification of the viscous force experienced within the fluid flow. This heightened viscous force functions as a resisting factor opposing the motion of the fluid, leading to a decrease in the flow field across all orientations. **However, it is evident that the power law index has a clear effect on the temperature distribution, as it directly affects the energy equation. In contrast, the power law index has an indirect effect on the concentration field, which leads to its minimal and insignificant effect on the temperature distribution.**

**Figure 3:**
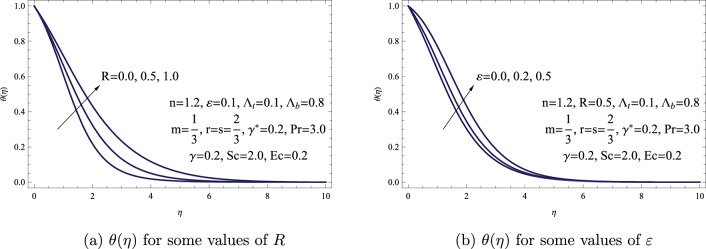
(**a**) $\theta (\eta )$ for some values of *R*. (**b**) $\theta (\eta )$ for some values of $\varepsilon$.

Figure 3 depicts how $\theta (\eta )$ is influenced by changes in the radiation *R* and thermal conductivity $\varepsilon$ parameters. It’s evident that when both *R*, and $\varepsilon$ are raised, both the thickness of the temperature boundary layer and the temperature distribution experience an increase. Physically, elevated values of both radiation and thermal conductivity parameters serve to improve the ability of the fluid to handle heat, ultimately resulting in a conspicuous rise in its temperature. This means that increased efficiency in both radiation and thermal conductivity contributes significantly to the fluid’s enhanced heat management, leading to a discernible escalation in its overall temperature. Moreover, the influence of thermal radiation on the mechanisms of heat and mass transfer can be substantiated by referring to pertinent and significant previously published papers outlined in references (^37–39^).

**Figure 4:**
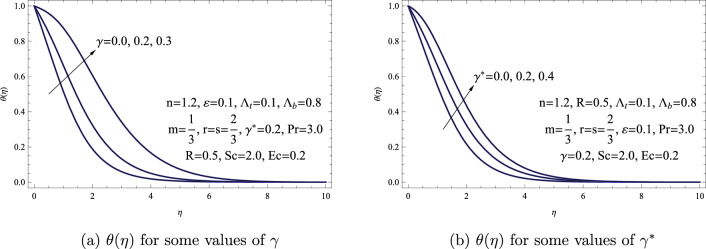
(**a**) $\theta (\eta )$ for some values of $\gamma$. (**b**) $\theta (\eta )$ for some values of $\gamma ^{*}$.

Figure 4 illustrates the effect of $\gamma$, and $\gamma ^{*}$ on the dimensionless temperature $\theta (\eta )$. It is evident that when both $\gamma$, and $\gamma ^{*}$ are positive, the heat generation results in a rise in temperature across the entire boundary layer. Additionally, it is evident that the influence of the temperature-dependent heat generation parameter on $\theta (\eta )$ is considerably greater than that of the spatially varying heat generation parameter. Abel et al.^40^ have reported similar outcomes concerning $\gamma$ and $\gamma ^{*}$, affirming the accuracy of our findings.

**Figure 5:**
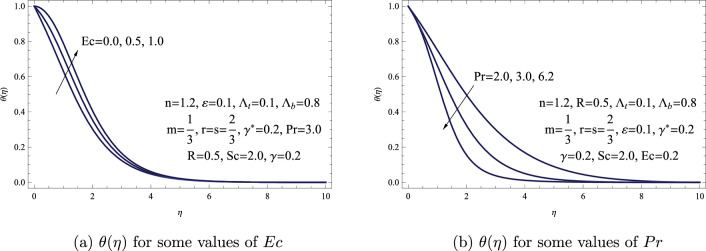
(**a**) $\theta (\eta )$ for some values of *Ec*. (**b**) $\theta (\eta )$ for some values of *Pr*.

Figure 5a gives $\theta (\eta )$ in the boundary layer region for a range of *Ec*. In contrast to the scenario without viscous dissipation, it’s noticeable that the dimensionless temperature rises with an increase in Eckert number *Ec*. The enhancement in fluid temperature caused by frictional heating is noted to be more prominent for elevated values of *Ec*, as anticipated. Figure 5b shows the effect of *Pr* on $\theta (\eta )$. This figure displays a declining trend in temperature distribution and the thickness of the thermal boundary layer as the Prandtl number enhances. Physically, in the PLF, a higher Prandtl number indicates a lower thermal conductivity. Wall heat transfer increases as a result of this decrease in thermal conductivity, which also lessens conduction. The power-law fluid’s ability to conduct heat is essentially reduced when the Prandtl number is raised, which amplifies the heat transfer at the fluid-solid interface.

**Figure 6:**
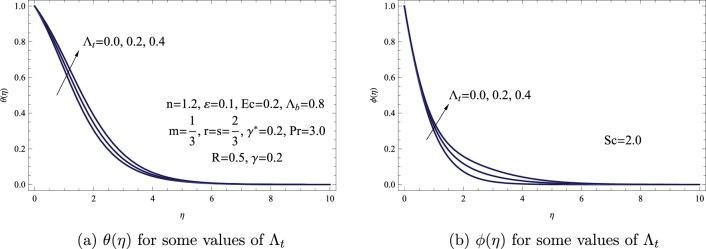
(**a**) $\theta (\eta )$ for some values of $\Lambda _{t}$. (**b**) $\phi (\eta )$ for some values of $\Lambda _{t}$.

The depictions of various thermophoresis parameters, denoted as $\Lambda _{t}$, regarding $\theta (\eta )$ and $\phi (\eta )$ distributions can be observed in Fig. 6. This figure suggests that as the thermophoresis parameter rises, there is an expected increase in both the concentration and temperature distributions. Physically, the heat transfer coefficient linked to the fluid is directly linked to the thermophoresis parameter **in a proportionally** manner. Hence, in the presence of a temperature gradient within the particle system’s flow area, smaller particles tend to spread more rapidly in hotter zones and at a slower pace in colder sections. This differential dispersion leads to an overall movement of particles from warmer to cooler areas. This migration outcome leads to the buildup of particles, causing higher particle concentrations within the colder portions of the particle mixture.

**Figure 7:**
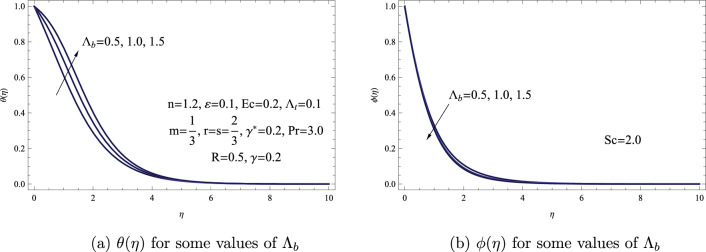
(**a**) $\theta (\eta )$ for some values of $\Lambda _{b}$. (**b**) $\phi (\eta )$ for some values of $\Lambda _{b}$.

Figure 7 illustrates the impacts of $\Lambda _{b}$ on the distributions of both concentration $\phi (\eta )$ and temperature $\theta (\eta )$. The information depicted in this figure clearly indicates that as the Brownian motion parameter increases, the concentration distribution of the fluid decreases. Conversely, the temperature field exhibits the opposite pattern. Physically, this phenomenon results from the fact that the collective effects of fluid molecules colliding with the particle surfaces are what fundamentally cause the Brownian motion of the particles. Furthermore, high values of the parameter function to impede the diffusion of nanoparticles in the fluid at distances from the surface, which lowers the dispersion or spread of concentration.

**Table 2:** Values of $Re_{x}^{\frac{-1}{n+1}} Sh_{x}$, $Re_{x}^{\frac{-1}{n+1}} Nu_{x}$ and $\frac{1}{2}Re_{x}^{\frac{1}{n+1}}Cf_{x}$ for various values of $n, R, \varepsilon , \gamma , \gamma ^{*},$ $Ec, Pr, \Lambda _{t}$, and $\Lambda _{b}$ with $Sc=2.0, m=\frac{1}{3}$, and $r=s=\frac{2}{3}$.

*n*	*R*	$\varepsilon$	$\gamma$	$\gamma ^{*}$	*Ec*	*Pr*	$\Lambda _{t}$	$\Lambda _{b}$	$\frac{1}{2}Re_{x}^{\frac{1}{n+1}}Cf_{x}$	$\frac{Re_{x}^{\frac{-1}{n+1}} Nu_{x}}{(1+R)}$	$Re_{x}^{\frac{-1}{n+1}} Sh_{x}$
0.7	0.5	0.1	0.2	0.2	0.2	3.0	0.1	0.8	0.768059	0.279378	1.04090
1.0	0.5	0.1	0.2	0.2	0.2	3.0	0.1	0.8	0.677696	0.274849	1.03038
1.2	0.5	0.1	0.2	0.2	0.2	3.0	0.1	0.8	0.636029	0.261189	1.02017
1.2	0.0	0.1	0.2	0.2	0.2	3.0	0.1	0.8	0.636029	0.265060	1.02330
1.2	0.5	0.1	0.2	0.2	0.2	3.0	0.1	0.8	0.636029	0.261189	1.02017
1.2	1.0	0.1	0.2	0.2	0.2	3.0	0.1	0.8	0.636029	0.239985	1.01924
1.2	0.5	0.0	0.2	0.2	0.2	3.0	0.1	0.8	0.636029	0.295616	1.01744
1.2	0.5	0.2	0.2	0.2	0.2	3.0	0.1	0.8	0.636029	0.227158	1.02280
1.2	0.5	0.5	0.2	0.2	0.2	3.0	0.1	0.8	0.636029	0.122618	1.03035
1.2	0.5	0.1	0.0	0.2	0.2	3.0	0.1	0.8	0.636029	0.473281	1.00359
1.2	0.5	0.1	0.2	0.2	0.2	3.0	0.1	0.8	0.636029	0.261189	1.02017
1.2	0.5	0.1	0.3	0.2	0.2	3.0	0.1	0.8	0.636029	0.063584	1.03099
1.2	0.5	0.1	0.2	0.0	0.2	3.0	0.1	0.8	0.636029	0.440750	1.00557
1.2	0.5	0.1	0.2	0.2	0.2	3.0	0.1	0.8	0.636029	0.261189	1.02017
1.2	0.5	0.1	0.2	0.4	0.2	3.0	0.1	0.8	0.636029	0.076553	1.03516
1.2	0.5	0.1	0.2	0.2	0.0	3.0	0.1	0.8	0.636029	0.324640	1.01448
1.2	0.5	0.1	0.2	0.2	0.5	3.0	0.1	0.8	0.636029	0.165594	1.02875
1.2	0.5	0.1	0.2	0.2	1.0	3.0	0.1	0.8	0.636029	0.005134	1.04316
1.2	0.5	0.1	0.2	0.2	0.2	2.0	0.1	0.8	0.636029	0.221093	1.01940
1.2	0.5	0.1	0.2	0.2	0.2	3.0	0.1	0.8	0.636029	0.231189	1.02017
1.2	0.5	0.1	0.2	0.2	0.2	6.2	0.1	0.8	0.636029	0.244238	1.02710
1.2	0.5	0.1	0.2	0.2	0.2	3.0	0.0	0.8	0.636029	0.275598	1.02764
1.2	0.5	0.1	0.2	0.2	0.2	3.0	0.2	0.8	0.636029	0.247806	1.01401
1.2	0.5	0.1	0.2	0.2	0.2	3.0	0.4	0.8	0.636029	0.223747	1.00438
1.2	0.5	0.1	0.2	0.2	0.2	3.0	0.1	0.5	0.636029	0.356354	1.00249
1.2	0.5	0.1	0.2	0.2	0.2	3.0	0.1	1.0	0.636029	0.212541	1.02480
1.2	0.5	0.1	0.2	0.2	0.2	3.0	0.1	1.5	0.636029	0.129750	1.02886

Table 2 displays the numerical data for the local skin friction coefficient (LSFC) $\frac{1}{2}Re_{x}^{\frac{1}{n+1}}Cf_{x}$, local Sherwood number (LSN) $Re_{x}^{\frac{-1}{n+1}} Sh_{x}$, and local Nusselt number (LNN) $Re_{x}^{\frac{-1}{n+1}} Nu_{x}$. These values are presented for various combinations of parameters including $n, R, \varepsilon , \gamma , \gamma ^{*}, Ec, Pr,$ $\Lambda _{t}$, and $\Lambda _{b}$. Observing the data in the table, it becomes apparent that increasing *n* leads to an overall decrease in the values of the LNN, LSN, and LSFC. Furthermore, the same table indicates that the local heat and mass transfer rates decrease when the thermophoresis parameter takes on larger values. It can be observed that higher values of both the heat generation parameter and the spatially varying heat generation parameter result in a reduction of the LNN, while they lead to an improvement in the LSN. This phenomenon arises because the heat generation mechanism elevates the fluid temperature in proximity to the surface. Consequently, the temperature gradient at the surface diminishes, ultimately causing a decrease in heat transfer at the sheet. Additionally, it can be determined that variations in the thermal conductivity parameter and Eckert number indices lead to only slight decreases in the Nusselt number.

In Table 3, we evaluated the residual error function (REF)^41^ of the present technique with the values of parameters $n=1,\,m=1/3,\,r=s=2/3,\,R=0.5,$ $\,\varepsilon =\Lambda _{t}=0.1,\, Pr=3.0,\,Sc=2.0,\,\gamma =Ec=\gamma ^{*}=0.2,\,\Lambda _{b}=0.8,$ and $N=7$. These values show the thoroughness of the proposed technique in this paper and confirm that this technique gives better accuracy.

**Table 3:** Values for the REF in the present technique.

$\eta$	REF of $f(\eta )$	REF of $\theta (\eta )$	REF of $\phi (\eta )$
0.0	3.951753E−07	4.756542E−08	8.014712E−06
1.0	4.753951E−08	3.852035E−07	5.985214E−08
2.0	9.014740E−08	2.852014E−07	2.014782E−08
3.0	2.963258E−06	9.015975E−08	0.963258E−08
4.0	7.023987E−08	3.321987E−08	1.654123E−07
5.0	4.753654E−08	7.456852E−06	4.014736E−06
6.0	5.741933E−06	0.854560E−08	7.654258E−07
7.0	0.321047E−08	9.014785E−07	3.011563E−08
8.0	9.852012E−08	7.852014E−08	5.456852E−07
9.0	3.741230E−08	5.951023E−07	9.759512E−08
10.0	1.756542E−06	0.987123E−08	6.550086E−06

## Conclusions

A study was conducted to investigate the heat and mass transfer characteristics of power-law nanofluid flow over a nonlinear stretching sheet, with various parameters being analyzed. The non-Newtonian nanofluid flow over a nonlinear stretching sheet was influenced by factors such as non-uniform heat generation, viscous dissipation, thermal radiation, and thermal conductivity effects. By employing similar transformations, the governing equations for energy, momentum, and concentration are converted into ODEs. The numerical solution to the transformed equations is obtained using the SCM with shifted VLPs, and a comprehensive analysis of the results is presented, considering various parameters related to the power-law fluid. Several conclusions can be drawn from the analysis, which are summarized as follows:

1. To reduce the energy distribution of the power-law nanofluid flow, it is necessary to utilize a fluid with a high *Pr* and decrease *n*.
2. Increasing the thermophoresis parameter and the power-law index, while simultaneously reducing the Brownian motion parameter, leads to an elevation in the concentration of the power-law nanofluid flow.
3. Elevating the temperature-dependent heat generation, Eckert number, space-dependent heat generation, and radiation parameter result in an increase in temperature. Conversely, an increase in the power-law index leads to a decrease in velocity.
4. The LNN, and LSN exhibit a declining trend as the power-law index, radiation parameter, and thermophoresis parameter increase.
5. The concentration profile responds oppositely to changes in $\Lambda _{b}$ compared to the thermophoresis parameter, while both parameters yield a similar impact on the temperature field.
6. The future research direction, building upon the foundation laid by this paper, involves investigating the flow of a hybrid power-law nanofluid subjected to the influences of Ohmic heating and variable density.

## Data Availability

The data sets used and analyzed during the present study are available from the corresponding author upon reasonable request.

## Abbreviations

$A^{*}, B^{*}$: Coefficients of the space and temperature-dependent heat generation
*c*: Constant
*C*: Concentration of fluid
$$C_{\infty }$$: Ambient concentration
$C_{w}$: The fluid’s concentration adjacent to the sheet
$c_{p}$: Heat capacity per unit mass
$Cf_{x}$: Local skin friction coefficient
$D_{B}$: Coefficient of diffusion
$D_{T}$: The coefficient associated with thermophoresis
*Ec*: The factor of viscous dissipation
*f*: Stream function with no dimensions
$$k^{*}$$: Absorption coefficient
*m*: Stretching parameter
*n*: Power-law parameter
$Nu_{x}$: Coefficient of heat transfer
*Pr*: Prandtl number
$q_{r}$: Radiative heat flux
$q^{'''}$: Non-uniform heat generation
$r,\,s$: Constants related to the temperature and concentration respectively beside the sheet
*R*: Radiation parameter
*Re*: Dimensionless Reynolds number
$Sh_{x}$: Coefficient of mass transfer
*Sc*: Schmidt number
*T*: Fluid temperature
$T_{\infty }$: Temperature of the fluid at a considerable distance from the surface
$T_{w}$: Temperature beside sheet
$$U_{w}$$: Stretching velocity
*u*, *v*: Components of the velocity vector in $x-$ and $y-$ directions; respectively

## Greek symbols

$$\rho $$: Fluid density
$$\psi $$: Stream function
$$\eta $$: Similarity variable
$$\Lambda _{t}$$: Thermophoresis parameter
$\Lambda _{b}$: Brownian motion parameter
$\kappa ,\,\kappa _{\infty }$: Thermal conductivity and Ambient thermal conductivity, respectively
$\theta$: Dimensionless fluid temperature
$\tau$: The ratio of the heat capacity of the nanomaterial to that of the fluid
$$\varepsilon $$: Conductivity parameter
$\gamma$: Temperature-dependent heat generation parameter
$\mu$: Viscosity of the fluid
$\gamma ^{*}$: Space-dependent heat generation parameter
$\phi$: Dimensionless fluid concentration
$$\nu $$: Kinematic viscosity

## Superscripts

*w*: Sheet condition
$\prime$: Derivative with respect to $\eta$
$\infty$: Condition away the sheet
